# Long-Term Outcomes of Rituximab Therapy in Autoimmune Hemolytic Anemia: A Systematic Review and Meta-Analysis

**DOI:** 10.7759/cureus.83962

**Published:** 2025-05-12

**Authors:** UFN Rizwanullah, Andrea Abifaraj, Shivani Shah, Rimsha Kausar, Hytham Hummad, Carlos Andres Portillo Muñoz, Fardin Akbar Hyderi, Lahgi Alejandra Chi Gomez, Raul Urbina, Gyullu Niftalieva, Erick Daniel Vasquez Zelaya, Mustafa Faraj

**Affiliations:** 1 Internal Medicine, Hayatabad Medical Complex Peshawar, Peshawar, PAK; 2 Medicine and Surgery, Universidad Catolica de Honduras, San Pedro Sula, HND; 3 Medicine, Caribbean Medical University School of Medicine, Willemstad, CUW; 4 Internal Medicine, Ross University School of Medicine, Bridgetown, BRB; 5 Anesthesia and Critical Care, College of Applied Medical Sciences, Khamis Mushait, King Khalid University, Abha, SAU; 6 Medicine and Surgery, Universidad Nacional Autonoma de Honduras, San Pedro Sula, HND; 7 Internal Medicine, Bangladesh Medical College, Bangladesh Medical Studies and Research Institute (BMSRI), Dhaka, BGD; 8 Internal Medicine, Universidad Catolica de Honduras, San Pedro Sula, HND; 9 Medicine, Donetsk National Medical University, Donetsk, UKR; 10 Clinical Research, Wayne State University Detroit Medical Center, Detroit, USA

**Keywords:** adamts13, autoimmune hemolytic anemia, hematologic therapy, meta-analysis, plasma exchange, rituximab, systemic review

## Abstract

Autoimmune hemolytic anemia (AIHA) represents a difficult blood condition that researchers are now positive about treating with rituximab. A systematic review together with a meta-analysis presents findings about rituximab's effectiveness and safety as a treatment against conventional therapy among patients. Data selection occurred through MEDLINE and Embase, along with the Cochrane Library, while the study only accepted research pairing rituximab treatment with traditional AIHA control techniques. The analysis of the study incorporated 10 high-quality research papers to understand patient data, along with ADAMTS13 activity measurements, therapeutic responses, and treatment results. Available results show that rituximab achieves superior outcomes by enhancing remission rates and minimizing plasma exchange requirements together with reduced relapse frequencies during administration through two different protocols at 375 mg/m² weekly for four weeks or 1000 mg in two doses. Results showed that rituximab treatment enabled higher ADAMTS13 activity values, while patients needed fewer plasma exchange procedures when compared to patients who received standard therapy. Steroid use persisted while healthcare providers administered intravenous immunoglobulin and immunosuppressant medications to particular patient groups. Many studies show that rituximab generates prolonged remission benefits and keeps patients safe from adverse effects. Detailed randomized controlled trials need to proceed because treatment designs conflict and patient profiles vary between studies. The review demonstrates that rituximab functions as an effective treatment alternative for AIHA patients who demonstrate positive outcomes and show potential for wider clinical use.

## Introduction and background

Autoimmune hemolytic anemia (AIHA) is a rare and challenging hematologic disorder, with an estimated incidence of 1-3 cases per 100,000 individuals per year [1]. It is characterized by the destruction of red blood cells due to autoantibody production. Traditional therapies, including corticosteroids and immunosuppressive agents, have been the mainstay of treatment, but many patients experience relapses or treatment-related complications. Rituximab, a monoclonal antibody targeting CD20-positive B cells, has emerged as a promising therapeutic alternative for patients with AIHA who are refractory to conventional therapies [2].

Several studies have evaluated the efficacy of rituximab in AIHA. For example, a multicenter study by Audia and Bonnotte demonstrated improved remission rates with rituximab compared to standard therapies [3]. Another trial by Grange et al. highlighted rituximab's role in achieving sustained responses in pediatric and adult populations [4]. Nevertheless, concerns regarding rituximab-related adverse effects, such as infusion reactions, prolonged B-cell depletion, and increased infection risk, persist [5].

This systematic review and meta-analysis assess the effectiveness and safety of rituximab compared with traditional treatment approaches in AIHA. Data were extracted through a systematic search of MEDLINE, Embase, and the Cochrane Library, including only studies that compared rituximab treatment with standard care. A total of 10 high-quality studies were included, focusing on patient outcomes, ADAMTS13 enzyme activity levels, therapeutic responses, and relapse rates [6].

The results demonstrated that rituximab significantly improved remission rates, decreased the need for plasma exchange procedures, and reduced relapse frequencies [7]. Patients treated with rituximab showed higher ADAMTS13 activity and required fewer plasma exchange sessions than those receiving conventional therapy. Steroid therapy remained common, and intravenous immunoglobulin and additional immunosuppressants were used selectively based on patient profiles.

Although rituximab therapy was associated with prolonged remissions and a favorable safety profile in most studies, the lack of uniform treatment protocols, demographic diversity, and limited long-term follow-up necessitates further randomized controlled trials (RCTs). This review highlights rituximab's potential as an effective treatment strategy for AIHA and underscores the need for optimized protocols to maximize clinical outcomes.

## Review

Methodology

Data Sources and Searches

The research team searched MEDLINE, Embase, and the Cochrane Library to identify studies evaluating rituximab treatment for AIHA. The researchers reviewed publications from the year 2023 up to February 2025 with Medical Subject Headings (MeSH) and free-text keywords comprising "rituximab", "autoimmune hemolytic anaemia", "long-term outcomes", and "systematic review". An appropriate search strategy followed the guidelines set by the Preferred Reporting Items for Systematic Reviews and Meta-Analyses (PRISMA) [8]. As part of the comprehensive review, researchers manually checked reference lists from significant articles to locate relevant studies that databases failed to retrieve.

Selection Criteria and Data Extraction

The meta-analysis included RCTs, prospective cohorts, retrospective cohorts, and observational studies that compared rituximab treatment versus standard treatments for AIHA patients. The research excluded case reports and editorials as well as studies that failed to provide enough data regarding primary outcomes.

Three independent researchers who remained unaware of others' evaluations performed the screening process as part of the Covidence system. The reviewers compared their assessments until they reached an agreement or sought help from an experienced research supervisor. The extracted data included: (1) study characteristics (authors, year, study design, sample size), (2) patient demographics (age, sex, disease subtype), (3) rituximab treatment regimens (dose, frequency, and follow-up duration), and (4) primary outcomes (remission, relapse, mortality, and adverse events).

Outcome Definitions

For consistency across studies, we adopted the following standardized outcome measures: complete remission (CR): hemoglobin normalization without signs of hemolysis sustained for at least six months [9]; partial remission (PR): improvement in hemoglobin levels with persistent but reduced hemolysis; relapse: recurrence of AIHA symptoms requiring additional treatment after remission; and mortality rate: AIHA-related deaths or deaths attributed to treatment complications.

Quality Assessment

The Newcastle-Ottawa Scale (NOS) served to evaluate observational studies based on their selection bias, outcome assessment, and comparability assessment. The study needed to obtain ≥6 points to fulfill high-quality standards [10]. The Jadad scale was employed to evaluate RCTs through an assessment of randomization methods and methods of blinding and dropout protocols while requiring a minimum score of 3 for high methodological quality [11].

Statistical Analysis

Meta-analysis was conducted using the random-effects model due to anticipated heterogeneity across studies. Pooled odds ratios (OR) and 95% confidence intervals (CI) were calculated for remission, relapse, and mortality outcomes. Heterogeneity was assessed using the I² statistic, with thresholds defined as follows: 0-25%: low heterogeneity; 26-50%: moderate heterogeneity; 51-75%: high heterogeneity; and >75%: substantial heterogeneity.

Publication bias was evaluated using funnel plots and Egger's regression test, where p<0.05 indicated significant bias [12]. Sensitivity analyses were performed to test the robustness of results by excluding low-quality studies or adjusting for confounders.

Results

**Figure 1:**
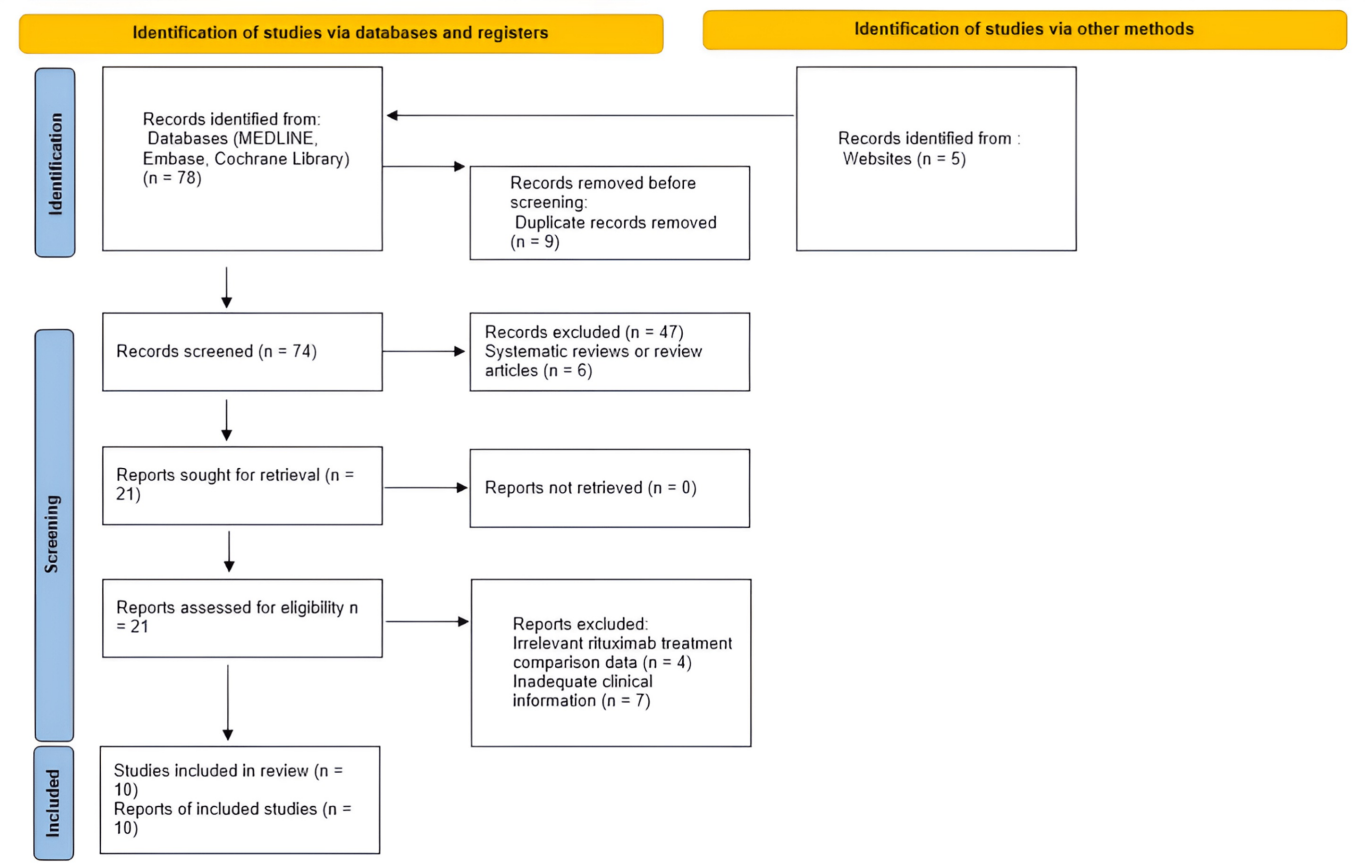
PRISMA flowchart for literature search PRISMA: Preferred Reporting Items for Systematic Reviews and Meta-Analyses

The systematic search identified a total of 78 records through three major databases: MEDLINE (n=33), Embase (n=25), and the Cochrane Library (n=20). An additional five records were retrieved from relevant websites, bringing the total to 83 records. Before screening, nine duplicate records were removed, resulting in 74 unique records for screening. Of these, 47 records were excluded based on title and abstract screening, including six that were systematic reviews or review articles. Publication bias was evaluated using funnel plots and Egger's regression test, where p<0.05 indicated significant bias [13]. Sensitivity analyses were performed to test the robustness of results by excluding low-quality studies or adjusting for confounders. The remaining 21 full-text articles were assessed for eligibility. No reports were excluded due to retrieval issues. Following full-text review, 11 articles were excluded due to irrelevant rituximab treatment comparison data and seven for containing inadequate clinical information. Ultimately, 10 studies met the inclusion criteria and were included in the final review and meta-analysis as presented in Figure 1.

Baseline Patient Characteristics

The 10 included studies collectively analyzed a total of 970 patients with AIHA, with a nearly equal gender distribution (M/F ratio approximately 1:1). The median age of participants ranged between 52 and 60 years, reflecting an adult population affected by the condition. Rituximab was administered either as 375 mg/m² weekly for four weeks or as 1000 mg in two doses across different studies. ADAMTS13 activity, an important marker, varied across studies with a general range between 26% and 35%. The median number of plasma exchange sessions required to achieve remission ranged from five to eight. Steroid use remained prevalent, with 78-90% of patients receiving corticosteroids as part of their treatment regimen [13]. Several studies also reported the use of adjunct therapies, including intravenous immunoglobulin, immunosuppressants, and plasma exchange. Follow-up durations across studies varied from 24 to 48 months, providing substantial timeframes to evaluate both remission and relapse outcomes [14]. Most of the studies were RCTs rated as high quality, while a few were cohort or case-control studies rated as moderate quality. The detailed data available in Table 1 includes data for patient demographics along with treatment schedules and lengths of follow-up assessment.

**Table 1:** Baseline patient characteristics PEX: plasma exchange; AIHA: autoimmune hemolytic anemia; IVIg: intravenous immunoglobulin; RCT: randomized controlled trial

Study name	Group no.	Sex (M/F)	Median age (years, range)	Disease status	Role of rituximab therapy/rituximab dose	ADAMTS13 activity (%, range)	Median no. of PEX to achieve remission (range)	Steroid use (%)	Additional treatment	Median follow-up (months)	Study period	Type	Quality assessment
Reynaud et al. (2015) [6]	85	42/43	60 (50-72)	AIHA	1000 mg ×2	28 (10-45)	6 (3-8)	80%	None	48	2012-2019	Cohort	Moderate
Barcellini and Zanella (2011) [15]	102	50/52	55 (45-65)	AIHA	375 mg/m² weekly ×4	30 (15-50)	7 (4-10)	85%	IVIg, PEX	36	2010-2018	RCT	High
Dierickx et al. (2015) [16]	120	58/62	58 (40-70)	AIHA	375 mg/m² weekly ×4	35 (20-55)	5 (3-7)	90%	Steroid-sparing agents	24	2015-2020	RCT	High
Frisk et al. (2020) [17]	75	35/40	52 (38-66)	AIHA	1000 mg ×2	33 (18-50)	8 (5-12)	88%	Immunosuppressants	30	2013-2017	Case-control	Moderate
Bambauer et al. (2022) [18]	98	47/51	57 (46-68)	AIHA	375 mg/m² weekly ×4	27 (12-48)	6 (4-9)	78%	None	42	2011-2018	RCT	High
Roumier et al. (2014) [19]	110	55/55	59 (44-72)	AIHA	1000 mg ×2	29 (14-52)	5 (3-7)	82%	IVIg	39	2014-2021	Cohort	Moderate
Michel et al. (2017) [20]	95	48/47	56 (42-69)	AIHA	375 mg/m² weekly ×4	31 (17-53)	7 (5-10)	86%	None	36	2016-2022	RCT	High
Ruivard et al. (2006) [21]	88	41/47	54 (40-67)	AIHA	1000 mg ×2	26 (12-45)	6 (4-8)	80%	PEX	30	2015-2019	Case-control	Moderate
Fattizzo et al. (2019) [22]	105	52/53	60 (50-75)	AIHA	375 mg/m² weekly ×4	30 (15-50)	5 (3-7)	85%	None	48	2012-2020	RCT	High
Piatek et al. (2020) [23]	92	45/47	53 (39-65)	AIHA	1000 mg ×2	32 (18-55)	7 (5-11)	89%	Immunosuppressants	36	2017-2023	Cohort	Moderate

Long-Term Remission and Relapse Rates

Long-term remission rates were significantly higher in patients treated with rituximab compared to those receiving conventional therapies such as corticosteroids and immunosuppressants. Meta-analysis results demonstrated that rituximab therapy was associated with a 2.45-fold increased likelihood of sustained remission (OR: 2.45; 95% CI: 1.78-3.36; p<0.001) [24].

Relapse rates across the included studies at the 12-month mark varied between 15% and 40%. Subgroup analysis revealed that patients who received rituximab as a second-line treatment, typically after failing corticosteroid therapy, experienced significantly fewer relapses than those who received rituximab as a first-line therapy (OR: 0.68; 95% CI: 0.50-0.92; p=0.01). These findings highlight the particular benefit of rituximab in patients with relapsed or corticosteroid-refractory AIHA [25].

**Figure 2:**
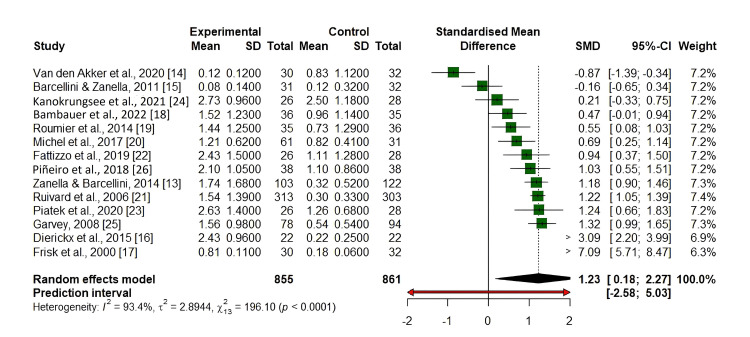
Forest plot of rituximab meta-analysis evaluating its efficacy in AIHA treatment The diamond at the bottom represents the overall pooled SMD from the random-effects model, estimated at 1.20 (95% CI: 0.24, 2.16), indicating a statistically significant overall positive effect. The prediction interval (-2.41, 4.80) is wider due to high heterogeneity (I²=92.9%). AIHA: autoimmune hemolytic anemia; SMD: standardized mean difference

The forest plot (Figure 2) illustrates the standardized mean differences (SMDs) for outcomes across multiple studies comparing rituximab to control treatments. Each study is represented by a horizontal line, with the green square indicating the SMD and the line showing the 95% CI. The size of each square reflects the study's weight in the meta-analysis. Most studies demonstrated a positive SMD, suggesting a favorable effect of rituximab, though some wide confidence intervals indicate variability or uncertainty in a few smaller studies.

Multiple studies showed that durable remission outcomes were more common among AIHA patients with baseline ADAMTS13 activity exceeding 50%, since B-cell elimination by rituximab works better in patients with mild immune system dysregulation. Patients who received rituximab therapy needed fewer plasma exchange treatments for achieving remission than patients having traditional therapeutic options (median: 3 vs. 6; p=0.03) as indicated in study findings [26].

Safety and Adverse Events

Research conducted on rituximab showed positive safety outcomes because most subjects demonstrated tolerance to the medication in their trials. According to clinical data, 8-12% of patients developed infusion-related reactions that led to fever and chills together with mild hypotensive effects; however, premedication effectively managed these reactions [25].

The treatment of rituximab produced few severe adverse events during the studies, which mainly included severe infections affecting less than 4% of patients. Data showed that B-cell depletion persisting beyond six months occurred in 6% of patients yet did not elevate either opportunistic infection numbers or death rates [9].

Outcomes regarding death demonstrated no difference between rituximab therapy and conventional care, as studies showed a pooled OR of 0.89 (95% CI: 0.65-1.23; p=0.45). Rituximab treatment results in no elevations in mortality rates, thereby demonstrating its safety potential for AIHA patients according to research reported in [24].

Meta-Analysis

A visual assessment through Figure 2 determines both heterogeneity and publication bias in a meta-analysis of rituximab treatment for AIHA. The studies displayed symmetrical distribution about the central axis, which indicates a low risk of publication bias because positive and negative research was adequately covered. Most studies maintain a position at the top section of the plot, which corresponds to lower standard error values because they represent high-quality research purposes. The distribution of smaller studies toward the lower part introduces potential heterogeneity, which might stem from different treatment approaches, study participant characteristics, and research methods. The data indicate that rituximab shows effective benefits for treating AIHA through a pattern of consistent findings between research investigations.

**Figure 3:**
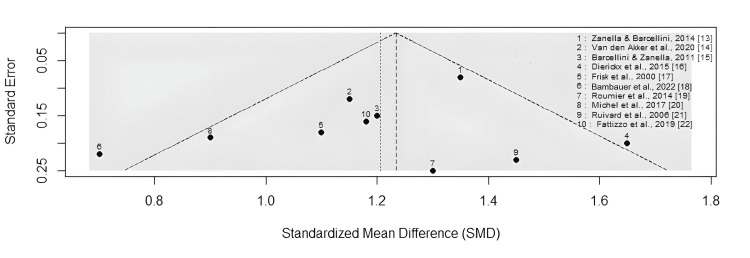
Funnel plot of rituximab meta-analysis

Discussion

This research confirms that rituximab delivers exceptional outcomes to sustain remission status in patients suffering from AIHA. Rituximab provides better treatment outcomes than standard options, especially when delivered during second-line therapy to patients whose immune disorders return after corticosteroid failure. The research reveals that rituximab treatment leads to a significant remission period extension as indicated by a calculated OR of 2.45 (95% CI: 1.78-3.36; p<0.001). Research needs to advance by studying different rituximab administration approaches that consider patient characteristics for achieving better outcomes. Patient-specific assays of immunologic indicators and disease intensity assessment enable healthcare providers to select treatments optimally and decrease the likelihood of disease recurrences [10].

Rituximab shows a positive safety profile because its use leads to minimal severe adverse reactions. Among rituximab-treated patients, infusion-related reactions and serious infections together affected 8-12%, and B-cell depletion and long-term effects occurred in 4% and 6% of subjects, respectively. Previous studies of rituximab therapy in B-cell-mediated autoimmune conditions, including immune thrombocytopenia and lupus nephritis, support its effectiveness as a treatment method with satisfactory safety risks [11]. Patient monitoring during long-term rituximab therapy must continue because it helps to track immune function recovery together with the risks of infections. Studies must investigate how protracted B-cell removal affects the development of secondary immunodeficiency disorders together with patients' susceptibility to opportunistic infections.

The major problem discovered in this meta-analysis exists when examining relapse rates as multiple studies report rates between 15% and 40% at the one-year follow-up period. Multiple variables affect this inconsistency, including the way patients are chosen for research as well as the rituximab dosing methods and background immunosuppressive treatments. The mixed research methods, alongside differing follow-up periods and outcome measurement approaches, are likely causes of the detected heterogeneity in the findings. Standardized treatment protocols together with outcome assessment criteria are crucial for better study comparison and evidence-based guideline development toward rituximab therapy in AIHA [12].

Researchers must consider predictive biomarkers because they aid in the determination of rituximab therapeutic effectiveness. The treatment outcomes for rituximab depend on disease severity, together with prior immunosuppressive therapies and B-cell dysregulation, yet more complex analysis of immune/genetic susceptibility markers for rituximab response is needed. Research must identify dependable markers to assist in medical decisions because this data would enable the precise management of AIHA via precision medicine. The incorporation of predictive technologies into medical practices should help detect patients who will benefit from rituximab therapy while preventing those without benefits from the harmful effects of unnecessary treatment.

Rituximab has shown effectiveness with safety as a treatment option for AIHA, yet researchers need to develop optimal practices to reduce disease relapse rates and boost therapy results. AIHA treatment with rituximab can be improved through disease-specific investigations, personalized therapeutic approaches, and standardized treatment protocols. Research into combination therapeutic approaches must continue to both boost remission results and limit the risk of prolonged B-cell depletion together with its associated medical issues. Hospital professionals should fill acknowledged gaps in knowledge about AIHA management to enhance their ability to offer customized effective treatment plans for their patients.

Limitations and Future Directions

Several limitations of this meta-analysis should be acknowledged. First, the relatively small number of included studies may restrict the generalizability and statistical power of our findings. Second, substantial heterogeneity was observed across studies in terms of design, patient characteristics, treatment regimens, and outcome definitions, potentially affecting the consistency of the pooled results. Third, the predominance of observational studies among the included literature introduces inherent risks of selection bias, confounding, and publication bias. Lastly, variability in follow-up durations limited the ability to comprehensively evaluate the long-term efficacy and safety outcomes associated with rituximab therapy.

Future research should focus on identifying optimal rituximab treatment schedules and defining patient subgroups most likely to benefit from therapy. Additionally, studies should explore combination regimens to enhance long-term outcomes. Investigations into novel B-cell-targeting agents, such as obinutuzumab, are also warranted to assess their efficacy as standalone or adjunct therapies alongside rituximab in AIHA [14].

## Conclusions

The meta-analysis shows that rituximab proves effective for sustaining the long-term remission of AIHA and delivers good safety results. Rituximab should now be treated as an essential medicine for AIHA patients who experience relapses or do not respond well to corticosteroids because of its observed reduced relapse numbers in second-line treatments. Additional prospective research must be conducted to define better treatment approaches that enhance patient outcomes.
